# Graphite-shell-chains selectively and efficiently produced from biomass rich in cellulose and chitin

**DOI:** 10.1038/s41598-020-69156-y

**Published:** 2020-07-22

**Authors:** Kyoko Suzuki, Yukie Saito, Noriyasu Okazaki, Tsutomu Suzuki

**Affiliations:** 1 Faculty of Engineering, Kitami Institute of Technology, 165 Koen-cho, Kitami, Hokkaido 090-8507 Japan; 2 Department of Global Agricultural Sciences, The Graduate School of Agricultural and Life Sciences, The University of Tokyo, 1-1-1 Yayoi, Bunkyo-ku, Tokyo, 113-8657 Japan

**Keywords:** Biotechnology, Materials science, Nanoscience and technology

## Abstract

Graphite-shell-chains have a worm-like nanocarbon configuration with a graphitic structure and mesopores, and they are easily produced from wood by using iron-group metal-catalysed carbonization at 900 °C. The simple production process with natural resources convinced us that this process may occur somewhere on Earth; the product of this process was indeed discovered as biogenic graphite by geochemists. However, the biogenic graphite was 3.7 billion years old, thus occurring long before wood appeared in the world. Here, we investigated appropriate carbon precursors other than wood in various materials and showed that carbon is selectively and efficiently obtained from biomass rich in cellulose and chitin. To enable selective and efficient production from this biomass, it seems the precursors provide a perfect amorphous carbon matrix where metal catalysts can reside at an active size to constantly create a graphite shell during carbonization. The results suggest that graphite-shell-chains could have existed in ancient times. Application developments of this biomass-derived nanocarbon will be useful for sustainable development goals.

## Introduction

**Figure 1:**
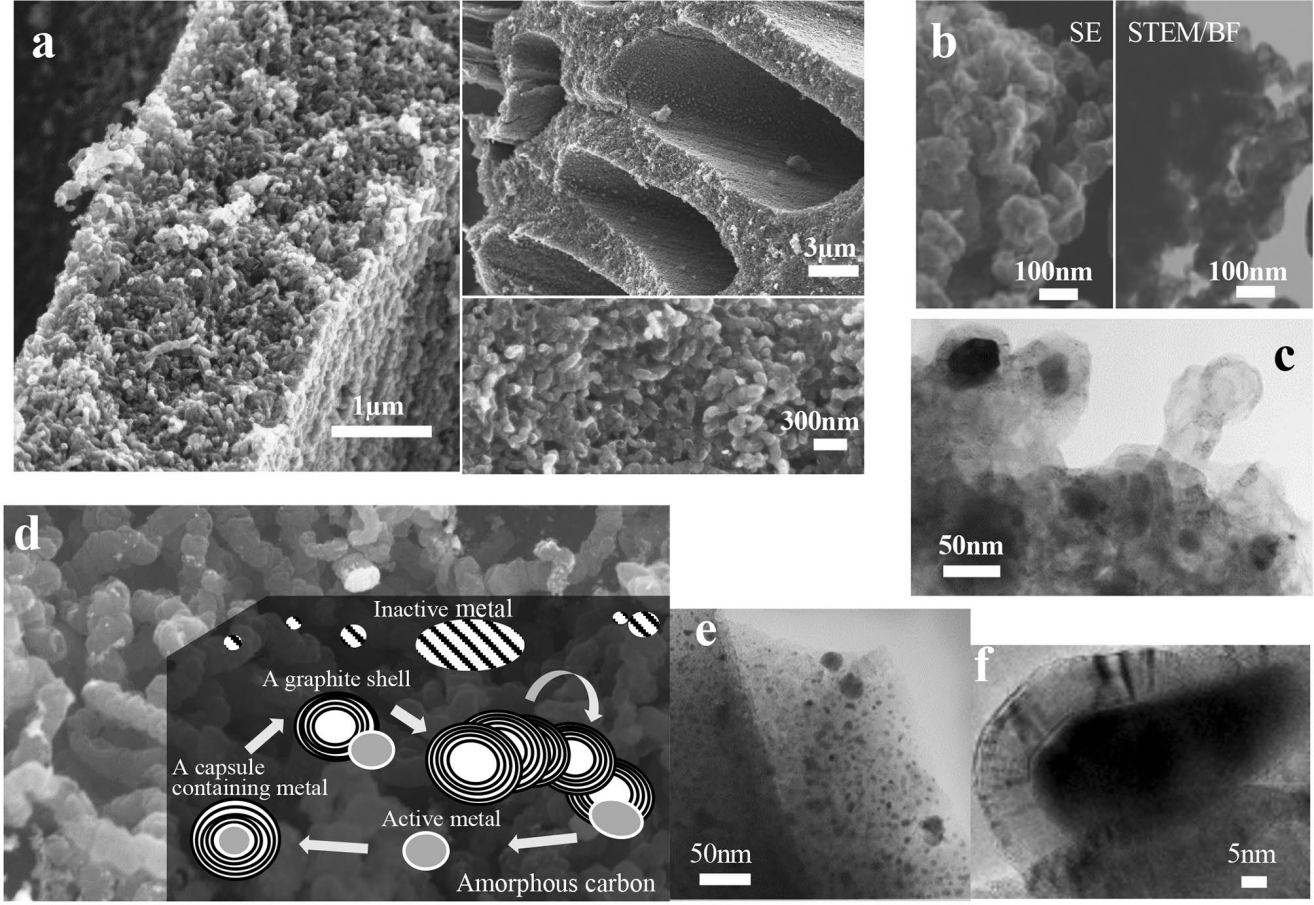
Electron microscopy images of graphite-shell-chains produced by iron-group metal-catalysed carbonization of wood and our proposed formation mechanism. (**a**) SEM images of graphite-shell-chains with iron showing their efficient formation in the wood cell wall. (**b**) STEM images of graphite-shell-chains with iron and their similarity to other images as evidence for the occurrence of biogenic graphite in early Archaean Isua metasedimentary rocks. (**c**) TEM image of graphite-shell-chains with iron without post-treatment showing that the active sizes of iron particles create capsules and graphite shells. (**d**) An illustration of our proposed formation mechanism with an SEM image of graphite-shell-chains with nickel. (**e**) TEM image of wood char by iron-catalysed carbonization at 500 °C showing iron particles dispersed in amorphous carbon just before graphitization. (**f**) TEM image of a graphite shell with iron showing an iron particle flowing out of a self-made capsule covered with curved graphite layers.

Graphite is a well-known carbon material that contributed to the Nobel prize winner in 2010 as a supply material of graphene and as a negative electrode material of lithium secondary ion batteries in 2019. It is an unevenly distributed mineral resource on Earth and is artificially made from coal at 3,000 °C. On the other hand, wood-derived carbon containing a certain amount of oxygen is a hard-graphitizing carbon, and its utilization is still limited. Under these circumstances, we produced a novel nanocarbon with a graphitic structure and mesopores ranging from 2–50 nm in diameter from wood by using iron-group metal-catalysed carbonization at 900 °C^1–3^. Because of the unique shape of the graphite shell with a diameter of 55–110 nm connected in a winding-chain formation, we first named it a worm-like carbon shell chain but later renamed it a graphite-shell-chain (GSC) (Fig. 1a, b, c)^4–6^.

Since GSCs are easily and efficiently obtained from various woods with 2–3 wt% iron-group metals by soaking the wood in a metal salt aqueous solution^7^, we expected that it would be naturally generated somewhere on Earth where wood with iron could be exposed to high heat (e.g., in contact with wildfires or volcanic magma). Two years after our submission^4^, geochemists used transmission electron microscopy (TEM) and scanning TEM (STEM) images to reveal biogenic graphite grains that were similar to GSCs, as shown in Fig. 1b, c^6,8^. However, the geochemists concluded that the grains were formed at least 3.7 billion years ago, thus occurring long before wood appeared in the world.

Currently, we are investigating various waste biomass and plastics for appropriate carbon precursors other than wood for practical applications of GSCs. In conducting the survey, we considered our proposed formation mechanism of GSCs as follows: each iron-group metal has a unique active particle size to form a graphite shell by flowing out of a graphitized capsule that was previously generated from amorphous carbon according to a dissolution–precipitation mechanism^9,10^ at 800–900 °C. Under these maintained conditions, the metal particles will constantly form a graphite shell in which the carbons are connected together in a chain until there is no adjacent amorphous carbon (Fig. 1d, e, f)^6,11–13^. According to this mechanism, any substance that becomes amorphous carbon by carbonization can be a candidate precursor. However, it has become clear from previous studies^6^ that GSCs cannot be made from substances consisting of only hydrocarbons.

## Results and discussion

In this study, waste phenol resin powders, empty fruit bunch (EFB) fibres, the extraction residue of coffee beans, tofu refuse, and hard charcoals of teakwood were examined by using our simple procedure for iron-catalysed carbonization at 850 °C (see Methods for details).

**Figure 2:**
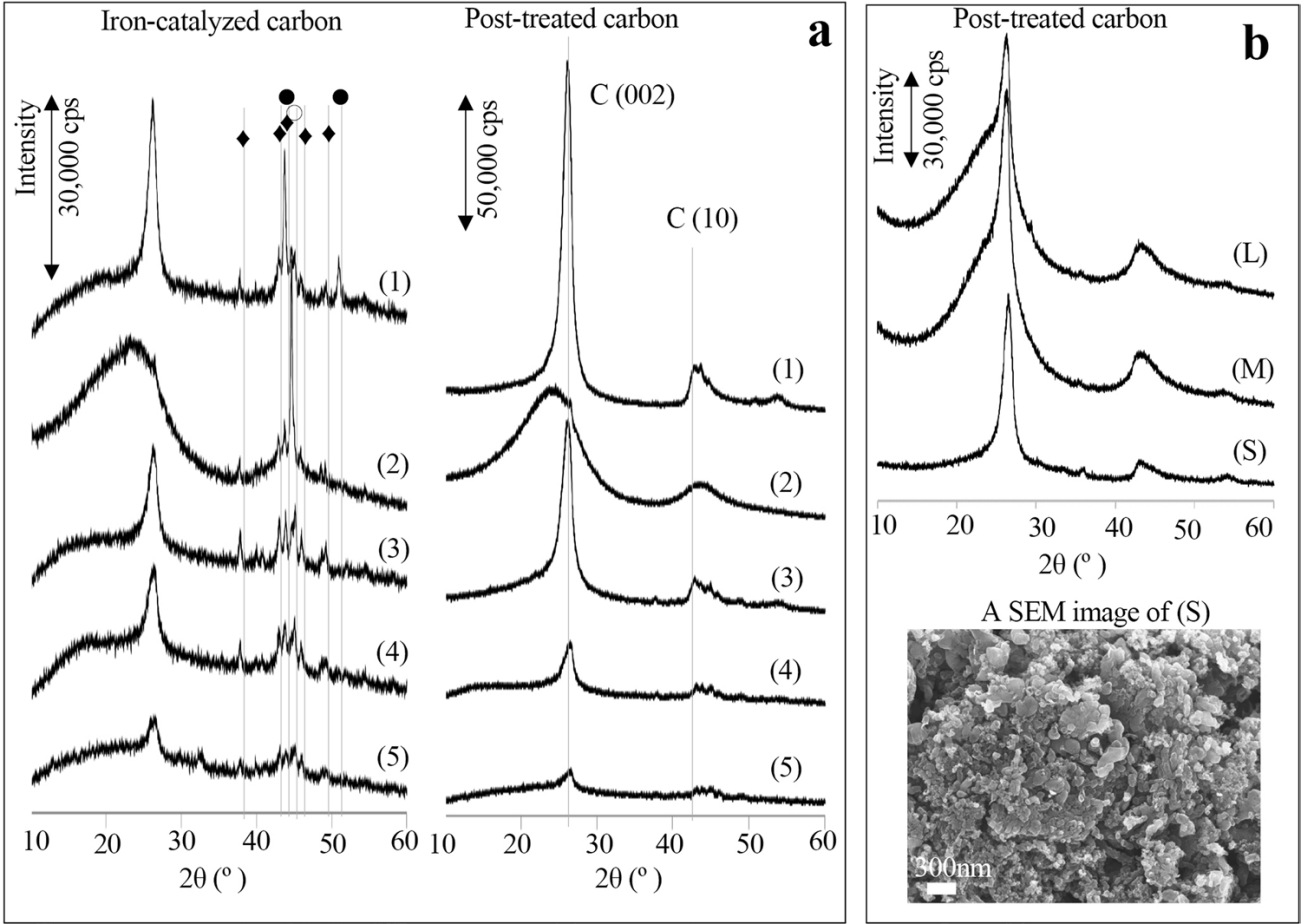
X-ray diffraction profiles and an SEM image of carbons produced by iron-catalysed carbonization and the post-treated carbon samples. (**a**) X-ray diffraction profiles of carbons produced by iron-catalysed carbonization (left) and the post-treated carbons (right) from wood (1), phenol resin (2), EFB fibre (3), the extraction residue of coffee beans (4) and Tofu refuse (5). Peaks from iron species are attributed to Fe_3_C (♦), α-Fe (○) and γ-Fe (●). (**b**) X-ray diffraction profiles of post-treated carbon samples produced by iron-catalysed carbonation from hard charcoals of teakwood with grain sizes of 2.8–5 mm (L), 2.8–1.7 mm (M) and 10 µ (S) showing that (S) can efficiently produce graphite-shell-chains. However, the SEM image shows that the graphite shell size is not as uniform as that of the wood-derived graphite shell.

In powder X-ray diffraction (XRD) analysis, the peak at a diffraction angle of approximately 26°, which is attributed to graphite (002), indicating the degree of carbon hexagonal network plane lamination, showed that biomass rich in cellulose selectively produced GSCs. The carbon from phenol resin seemed to form a trace amount of GSCs with α-Fe crystals too large to form a graphite shell (Fig. 2a). The results caused us to expect many hydroxyl groups in cellulose to enable the control of the metal particle size in the early stage of carbonization. However, the hard charcoal of teakwood carbonized at approximately 1,000 °C, with low porosity (the Brunauer–Emmett–Teller (BET) surface area was 94 m^2^/g) and few remaining hydroxyl groups, was also unexpectedly able to produce GSCs, as shown in Fig. 2b. Compared to that of GSCs formed from wood, the conversion efficiency of amorphous carbon from GSCs formed from the hard charcoal of teakwood strongly depended on the grain size, and the diameter of the shell observed by scanning electron microscopy (SEM) was not uniform, varying in the range of 10–150 nm. This suggests that the active size of iron particles seems to be standardized and maintained by a special amorphous carbon matrix made of cellulose or the component glucose rings.

To investigate their effects on GSC production, we chose starting materials including glucose and/or other rings similar to glucose, such as cellulose derived from absorbent cotton, sugar-derived sucrose that has a glucose ring and a fructose ring, glucomannan derived from devil's tongue powder that has a glucose ring and a mannose ring, and commercially available chitin that has a structure similar to cellulose except for the hydroxyl group at the 2-carbon position of cellulose in place of the acetamido group for chitin.

**Figure 3:**
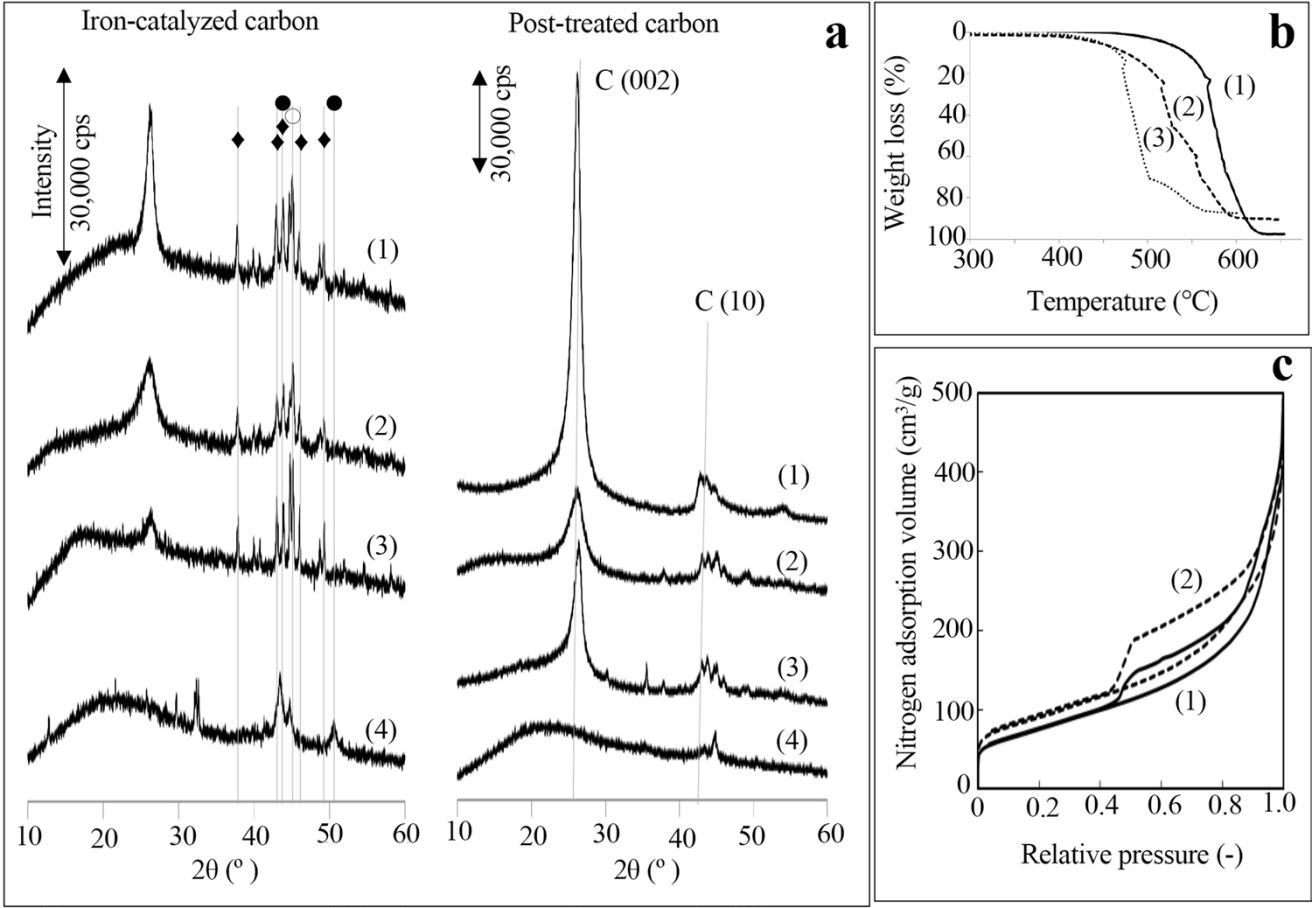
Characterization of carbons produced by iron-catalysed carbonization and the post-treated carbons from various precursors composed of glucose and similar rings. (**a**) X-ray diffraction profiles of carbons from cellulose (1), chitin (2), sucrose (3) and glucomannan (4) showing the inability of glucomannan (4) to produce graphite-shell-chains and hardness of iron removal for the carbon from sucrose (3). Peaks from iron species belong to Fe_3_C (♦), α-Fe (○) and γ-Fe (●). (**b**) Thermogravimetric curves of post-treated carbons from cellulose (1), chitin (2) and sucrose (3) showing that they have different thermochemical stabilities. (**c**) Nitrogen adsorption desorption curves of post-treated carbons from cellulose (1) and chitin (2) showing that chitin-derived carbon has more mesopore volume, as shown by the hysteresis of the curves, which indicates mesopore formation.

The XRD analysis in Fig. 3a showed that glucomannan experienced a kind of congelation during heating and could not produce any GSCs and Fe_3_C, which may have indicated the initial stage of catalytic graphitization. Cellulose-, sucrose- and chitin-derived carbon structures were aligned by the XRD intensity of the graphite (002) peak as if they were efficiently composed of GSCs in the order shown in Fig. 3a on the right. However, post-treated sucrose-derived carbon had the lowest thermochemical stability, with the largest amount of iron residue among the three carbon structures composed of GSCs (Fig. 3b). For post-treated chitin-derived carbon, the amount of iron residue was larger and the thermochemical stability was lower than those of cellulose-derived carbon. Moreover, despite having the lowest XRD peak for graphite (002) among the three carbons in Fig. 3a on the right, post-treated chitin-derived carbon had more mesopores, which likely reflect shell defects and voids between shells^6^, than those of cellulose-derived carbon (Fig. 3c).

**Figure 4:**
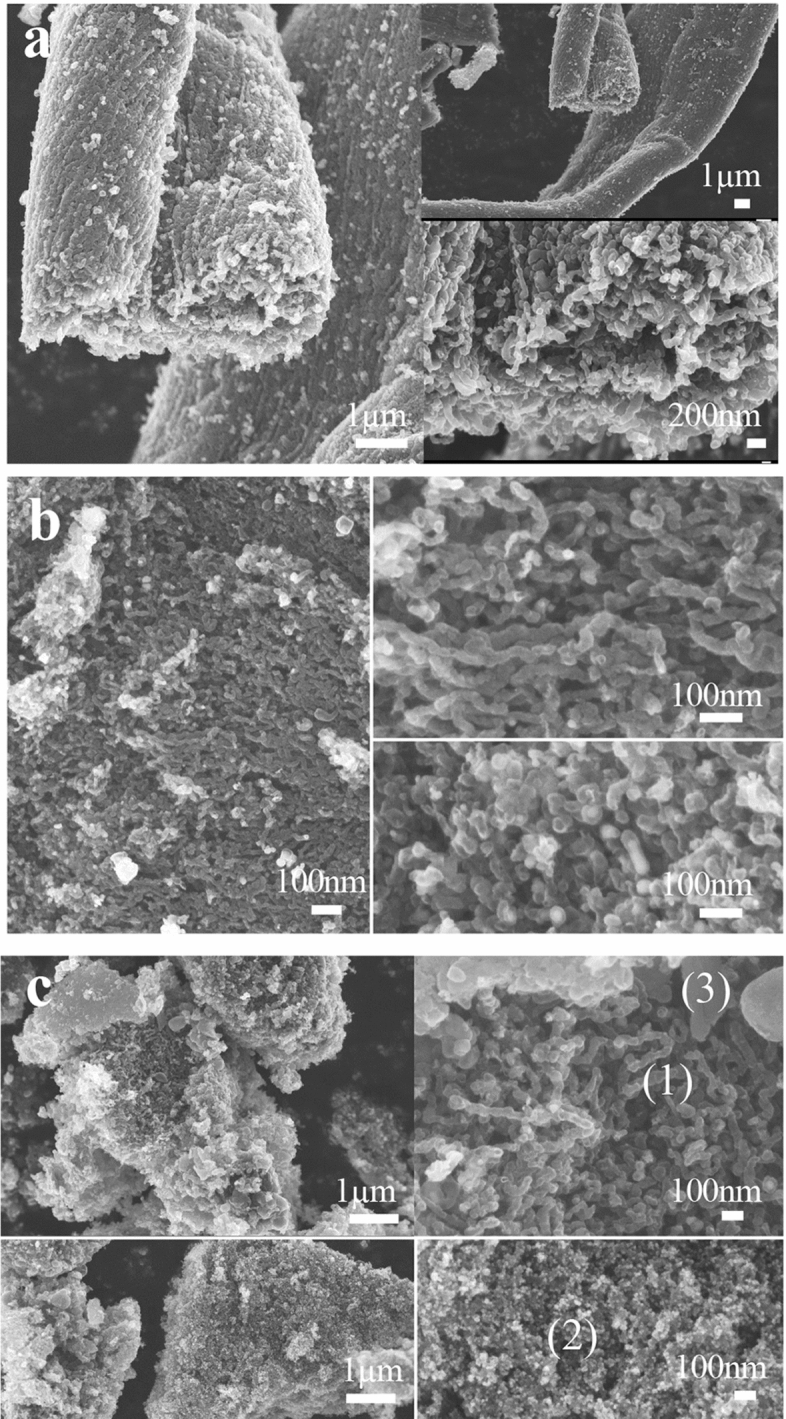
Scanning electron microscopy images of carbons, including graphite-shell chains. (**a**) SEM images of carbon from cellulose showing perfect production of graphite-shell-chains along the original cotton fibre twisting direction. (**b**) SEM images of carbon from chitin showing efficient production of graphite-shell chains along the original fibre swirling direction. Compared with the size of the cellulose-derived shell in (**a**) the average size is almost half, and some iron remaining in the shells is observed (right, lower part). (**c**) SEM images of carbon from sucrose showing partial production of graphite-shell-chains (1), small capsules of iron (2) and amorphous carbon blocks (3).

These results were fully explained by the following SEM observations. The cellulose perfectly produced GSCs along twisting cotton fibres, as shown in Fig. 4a. The GSC size was almost the same as the size of wood-derived GSCs^6^. Chitin also produced GSCs efficiently along the swirling fibre, although the average shell size was half that of the cellulose-derived graphite shell, and some iron residue was observed in the shell (Fig. 4b). In Fig. 4c, sucrose made three types of carbon: (1) a slightly smaller GSC than that from cellulose, (2) grains of carbon capsules containing Fe or Fe oxide, which are smaller than the graphite shells, and (3) amorphous carbon blocks. In other words, the difference in graphite shell sizes must have affected the height of the XRD peak for graphite (002); in addition, the iron residue in the GSCs from chitin and in the capsules from sucrose might result in low thermochemical stability, and the efficient production of small GSCs from chitin could induce rich mesopores.

This study reveals that among materials containing glucose rings and other similar rings, materials having a well-ordered structure, such as fibres with homogeneous rings, can produce GSCs particularly efficiently. It is also found that the size of GSCs is standardized by not only the metal species reported in other papers^6^ but also the precursors; thus, the size of the shell is determined by various combinations. The specificity of these results is not fully explained by previous theories for non-graphitic carbons, such as the cross-linking carbon structure and the inclusion of non-six-membered rings^14–17^, and requires further research on GSC formation.

However, the results that biomass rich in cellulose and chitin other than wood selectively and efficiently produces GSCs suggested that GSCs may have existed in ancient times long before wood appeared in the world. For example, chitin, the major constituent of fungal cell walls, was recently presented as evidence of the discovery of fungal fossils in an 810–715 million-year-old dolomitic shale rock^18^.

Many graphitic structures from woody biomass and thermosetting resins using iron-group metal-catalysed carbonization have been sporadically reported^19–29^. However, their shapes were not specified since most of these structures were partially formed in a solid carbon matrix, and their hard isolation from the matrix made them undetectable in SEM observations. Moreover, GSCs are composed of different shapes ranging from spherical to flat, various sizes as mentioned above, and chains with various numbers of connected shells and different connection distances between shells. These variations may cause researchers to conclude that they are different carbon structures formed by a different mechanism. This study clarifies that GSCs are efficiently produced from biomass rich in cellulose and chitin; therefore, the shape can be easily observed by SEM after the removal of the small amount of amorphous carbon deposited on the surface.

Thus, GSCs are a unique biomass-derived nanocarbon created from the earth and made at considerably lower temperatures than regular graphite. In addition to past works^3,7,23,26,29–31^, various utilizations should be developed by other researchers for sustainable development goals.

## Methods

### Materials

Sawdust from Japanese larch was used as a reference wood sample. Phenol resin powder and EFB fibre, kindly provided by manufacturers, the extraction residue of commercially available coffee beans, and commercially available Tofu refuse powder were used as raw materials. Hard charcoals of teakwood with variable particle sizes were also kindly provided by the manufacturer. As a representative substance of pure cellulose, we used commercially available medical absorbent cotton. Chitin was purchased from FUJIFILM Wako Pure Chemical Corporation. As a representative substance of sucrose, commercially available granulated sugar was used, and devil’s tongue powder, kindly provided by a food manufacturer, was used as a representative substance of glucomannan.

### Preparation of carbon samples

Various dried materials less than 1.4 mm in diameter were mainly used as raw precursors. The raw precursor was individually loaded with iron nitrate Fe(NO_3_)_3_·9(H_2_O) by the typical aqueous impregnation method for 24 h, and the loading amount was adjusted to 3 wt% as iron metal in the moisture-free precursor. For hard charcoal of teakwood, the loading amount was 10 wt% as iron metal. The dried iron-loading sample after excess water was evaporated was transferred by a stainless steel vessel and placed in a vertical stainless steel tube reactor. The reactor was then electrically heated downstream of nitrogen (1 mL STP cm^−2^ min^−1^) from room temperature to 850 °C at 10 °C min^−1^. After the temperature was maintained for 1 h, the tube reactor was cooled to room temperature by blowing air on the outside of the tube reactor. During the whole period, including the cooling step, the nitrogen flow into the reactor was continued. As the first post-treatment for the removal of iron, iron-loaded carbon was soaked in 1 M HNO_3_ with stirring at room temperature for 24 h and then thoroughly washed with distilled water and dried at 105 °C. As a second post-treatment, the iron-free carbon taken in a porcelain crucible was heated at 400 °C in a muffle furnace to reach 30% loss of the entire carbon in weight for selective and complete elimination of the amorphous carbon to leave GSCs.

### Characterization of carbon

Powder XRD with Cu-Kα radiation (Rigaku Ultima IV) was performed for the carbon samples with and without the post-treatments. Thermochemical stability of the carbon samples was evaluated by measuring their non-isothermal thermogravimetric (TG) curves (ULVAC TGD 9600) under the following conditions: a sample weight of 20 mg, a heating rate of 10 °C min^−1^, a maximum temperature of 700 °C, and an ambient flow of artificial air at 80 mL STP min^−1^. To determine the porosity of the carbon samples, nitrogen adsorption and desorption isotherms were measured at − 196 °C (ThermoQuest, Sorptomatic 1990). The surface state and morphology were observed by SEM (JOEL JSM-6701F), STEM (Hitachi SU8000 and S-5500) and TEM (JOEL Model-2000EX and FEI Titan 80–300).
